# Non-Anticoagulant Fractions of Enoxaparin Suppress Inflammatory Cytokine Release from Peripheral Blood Mononuclear Cells of Allergic Asthmatic Individuals

**DOI:** 10.1371/journal.pone.0128803

**Published:** 2015-06-05

**Authors:** Madhur D. Shastri, Niall Stewart, James Horne, Syed Tabish R. Zaidi, Sukhwinder Singh Sohal, Gregory M. Peterson, Heinrich Korner, Nuri Gueven, Rahul P. Patel

**Affiliations:** 1 Pharmacy, School of Medicine, Faculty of Health, University of Tasmania, Hobart, Tasmania, Australia; 2 Central Science Laboratory, University of Tasmania, Hobart, Tasmania, Australia; 3 Menzies Institute for Medical Research, Faculty of Health, University of Tasmania, Hobart, Tasmania, Australia; 4 Breathe Well Centre of Research Excellence for Chronic Respiratory Disease and Lung Ageing, School of Medicine, Faculty of Health, University of Tasmania, Hobart, Tasmania, Australia; Université Libre de Bruxelles, BELGIUM

## Abstract

**Background:**

Enoxaparin, a low-molecular-weight heparin, is known to possess anti-inflammatory properties. However, its clinical exploitation as an anti-inflammatory agent is hampered by its anticoagulant effect and the associated risk of bleeding.

**Objective:**

The aim of the current study was to examine the ability of non-anticoagulant fractions of enoxaparin to inhibit the release of key inflammatory cytokines in primed peripheral blood mononuclear cells derived from allergic mild asthmatics.

**Methods:**

Peripheral blood mononuclear cells from allergic asthmatics were activated with phytohaemag glutinin (PHA), concanavalin-A (ConA) or phorbol 12-myristate 13-acetate (PMA) in the presence or absence of enoxaparin fractions before cytokine levels were quantified using specific cytokine bead arrays. Together with nuclear magnetic resonance analysis,time-dependent and target-specific effects of enoxaparin fractions were used to elucidate structural determinants for their anti-inflammatory effect and gain mechanistic insights into their anti-inflammatory activity.

**Results:**

Two non-anticoagulant fractions of enoxaparin were identified that significantly inhibited T-cell activation. A disaccharide fraction of enoxaparin inhibited the release of IL-4, IL-5, IL-13 and TNF-α by more than 57% while a tetrasaccharide fraction was found to inhibit the release of tested cytokines by more than 68%. Our data suggest that the observed response is likely to be due to an interaction of 6-*O*-sulfated tetrasaccharide with cellular receptor(s).

**Conclusion and Clinical Relevance:**

The two identified anti-inflammatory fractions lacked anticoagulant activity and are therefore not associated with risk of bleeding. The findings highlight the potential therapeutic use of enoxaparin-derived fractions, in particular tetrasaccharide, in patients with chronic inflammatory disorders.

## Introduction

Low-molecular-weight heparins (LMWHs) are a heterogeneous mixture of structurally complex oligosaccharides [1]. In clinical practicethese molecules are widely used as anticoagulants but additionally, theyare reported to possess anti-inflammatory properties [2,3]. Enoxaparin, the most widely used LMWH, is known toinhibit T cell mediated release of multiple cytokines, such as IL-4, IL-5, IL-13 and TNF-α, involved in various inflammatory disorders including asthma [4]. A model for a chronic inflammatory disease and a potential target for an anti-inflammatory therapy is asthma, a complex multifactorial disorder of conducting airways. Its inflammatory pathology is characterised by reversible airway obstruction and airway hyper-responsiveness. It is estimated that approximately 300 million people worldwide suffer from asthma and it is associated with severe morbidity, and sometimes even mortality [5]. Inflammation in asthma is characterised by activation of T-helper type 2 (Th2) cells, production of immunoglobulin E (IgE) antibodies and eosinophilia [6]. Although T cells residing in the lungs (most of which are memory cells) are important for the local defence mechanism, excessive recruitment of T cells (mainly Th2 type) in the lungs, as well as their response to various allergens, is believed to be largely responsible for coordination of the inflammatory processes associated with allergic asthma [7]. In response to allergens, Th2 cells do not proliferate in the lungs but rather migrate into the lung from regional lymph nodes and then produce various inflammatory cytokines, such as interleukin (IL)-4, IL-5, IL-9, IL-13 and tumor necrosis factor (TNF)-α, and chemokines, such as monocyte derived chemokine, in response to the specific allergen [8]. IL-4 and IL-13 are normally involved in the process responsible for the production of IgE antibodies. IL-4 and IL-5 enhance the survival of eosinophils, resulting in eosinophilic inflammation. IL-13 (and IL-9 to some extent) is reported to be important for bronchial hyper-responsiveness [9]. On the other hand, TNF-α sustains lung inflammation by increasing the recruitment of neutrophils and eosinophils in the airways [10].

Inhaled corticosteroids are the most effective drugs currently available for the treatment of asthma [11]. They effectively control asthma in the majority of patients. However, apart from the fact that the prevalence of steroid-resistant asthma is rising [12], corticosteroids are associated with a number of problems. For example, the symptoms appear soon after the treatment is stopped and with long-term treatment they increase the risk of osteoporosis in adults and reduced bone growth in children [13,14]. Also, at recommended doses they have little or no effect on the process of asthma-associated lower airway remodelling [15]. An advanced understanding of the complex pathophysiology that drives this inflammatory disease has resulted in the development of new and emerging biological therapies, such as monoclonal antibodies, that are directed against a specific type of cytokine that is involved either in cell proliferation or the inflammatory response [16]. Apart from their cost and potentially severe side effects (such as hypersensitivity and susceptibility to various types of infections), monoclonal antibodies, at least for the treatment of asthma, have so far failed to meet initial theoretical expectations. It has been proposed that their limited clinical efficacy could be due to their inability to simultaneously inhibit multiple cytokines involved in the pathogenesis of asthma [17]. Therefore, the search for safer, cheaper and effective drugs for the treatment of asthma continues. Among these drugs, LMWHs have attracted much interest in the field of inflammation.

Several experimental and clinical studies have shown the beneficial effects of LMWHs in various types of inflammatory disorders, including asthma [18–20]. The precise mechanism(s) behind the anti-inflammatory effects of LMWHs are yet to be fully explored. However, it has been proposed that because of their high negative charge they can interact and modulate the activity of a wide range of biological molecules, including various types of immune cells and pro-inflammatory cytokines [21]. As the recruitment and activation of T cells in the lung is believed to be one of the central events in the initiation, progression and persistence of asthma, drugs (e.g. enoxaparin) that can suppress T cell-mediated inflammatory responses can be considered a promising therapeutic strategy [22]. However, like any other LMWH, enoxaparin is composed of both non-anticoagulant andanticoagulant components, referred to as oligosaccharides. Its potential use as an anti-inflammatory agent could be problematic due to the inherent risk of bleeding caused by the presence of anticoagulant oligosaccharides [21]. This problem can potentially be resolved by separating the anticoagulant fractions (oligosaccharides responsible for anticoagulant effect) and non-anticoagulant fractions (oligosaccharides potentially responsible for anti-inflammatory effect). However, separation of such a complex polysaccharide is a long-standing problem [23]. Nevertheless, we recently developed a novel chromatographic technique that allows the separation of different oligosaccharides of enoxaparin [24]. In the current study we isolated chromatographically separated oligosaccharides of enoxaparin and investigated their anticoagulant effect, as well as their ability to inhibit the T cell mediated release of important cytokines before postulating the potential mode of action alongside the structure activity relationship.

## Material and Methods

### Reagents

All chemicals and reagents, if not otherwise stated, were obtained from Sigma-Aldrich (NSW, Australia). The sodium salt of enoxaparin (100 mg/mL) was purchased from Aventis Pharma (NSW, Australia), deuterium oxide (D_2_O) was purchased from Cambridge Isotope Laboratories (Andover, MA, USA), anti-factor Xa (AFXa) kit was purchased from American Diagnostica (Stamford, CT, USA) and fetal bovine serum was purchased from Invitrogen (NY, USA).

### Recruitment of Participants

Five healthy (mean age: 33.8 years, range: 26–48 years; 4 males, 1 females) and ten allergic asthmatic subjects (mean age: 40.9years, range 19–59 years; 4 males, 6 females) were recruited by invitation. The healthy volunteers were not suffering from any acute or chronic diseases and the asthmatic subjects were suffering from no other diseases apart from intermittent asthma exacerbated by allergies. Exclusion criteria were use of systemic or inhaled corticosteroids or any other immunomodulatory medications within two months prior to blood sampling. No information of either the use of other medications or forced expiratory volume (FEV1) was obtained from the recruited participants.

### Ethics Statement

The research protocol was approved by the Health and Medical Human Research Ethics Committee (Tasmania, Australia) Network (Approval number: H0013117). Written informed consent for the collection of blood samples was obtained from the recruited subjects.

### Isolation of Peripheral Blood Mononuclear cells (PBMCs) from Whole Blood

Whole blood (80 mL) from each subject was collected before PBMCs were isolated. Isolation of PBMCs was conducted by standard methods via density separation using 30 mL of blood aliquots layered onto 20 mL of Histopaque. Following centrifugation at 200g for 30 minutes at 20°C (Eppendorf; Model: 5810R), PBMCs were aspirated from the Histopaque/aqueous interface and centrifuged at 700g for 10 minutes. Cells were washed twice with serum-free media and resuspended in complete medium [RPMI-1640 supplemented with 2.0 mM L-glutamine, 10% fetal bovine serum and antibiotics (penicillin G and streptomycin)].

### Separation and Isolation of Enoxaparin Fractions

Chromatographic separation of enoxaparinwas carried out using a previously described ion-exchange chromatography (IC) method [24]. Briefly, enoxaparin separation was performed on a semi-preparative Dionex CarboPac PA100 (250 mm, 9 mm ID, 8.5 μm) strong anion-exchange column (Thermo Fisher Scientific, NSW, Australia). Mobile phases were composed of Milli-Q water (A) and 2 M NaCl (B). The linear NaCl elution gradient (mobile phase B; 32 to 74%) was used over 70 minutes with a constant flow rate of 2.0 mL/minute. A column temperature of 40°C was combined with UV detection at 232 nm.

### Collection and Desalting of ICSeparated Enoxaparin Fractions

IC separated enoxaparin fractions were collected and desalted as described previously [24] with minor modifications. Briefly, collected fractions were concentrated on a miVac DNA centrifugal concentrator (Genevac Ltd, Suffolk, UK) at 40°C and subsequently desalted using PD MidiTrap G-10 columns (GE Healthcare Life Sciences, Uppsala, Sweden). The desalted fractions were kept at 4°C until use. The concentration of IC separated fraction of enoxaparin was determined by constructing a calibration curve from the peak areas of LMWH standards against their known concentrations. The recovery of each desalted fractionwas calculated using the differences in the peak areas of the desalted fraction and enoxaparin fraction eluted at the same time. Each desalted fraction was tested for its anticoagulant activity and its inhibitory effect on cytokine release from activated PBMCs.

### Determination of NaCl Content

The NaCl content in the desalted fractions of enoxaparin was determined using flame photometry as described previously [24]. Briefly, a stock solution of NaCl (1 mg of sodium/mL) was prepared by dissolving 2.54 g of NaCl in one litre of Milli-Q water. The stock solution was serially diluted to prepare different standard sodium solutions at concentrations of 5 to 50 ppm. The standard solutions (n = 3) were introduced into a low temperature, single channel flame photometer (Model-PFP7, VWR International, Murarrie, Australia) and emission intensities were measured at 589 nm. The concentration of NaCl in the desalted enoxaparin fractions (n = 3) was calculated using the standard sodium calibration curve.

### Analysis of Anticoagulant Activity

The potentiating effect of IC-derived enoxaparin fractions on anti-thrombin III inhibition of activated factor Xa was determined using a previously described low-volume microtitre plate anti-factor Xaassay [25]. Briefly, a solution containing anti-thrombin III, FXa and IC-derived enoxaparin fractions was incubated for 3 minutes at 37°C. FXa substrate was added immediately and the solution was incubated for another 10 minutes. The reaction was quenched using glacial acetic acid and the intensity of developed colour was spectrophotometrically measured at 405 nm (Multiskan Go, SkanIt software, ThermoFisher Scientific).

### Preparation of Stock Solutions for PBMC Culture Treatments

Stock solutions of enoxaparin fractions at 1 mg/mL were prepared in RPMI-1640 medium and filter sterilized through 0.2 μm pore size syringe filters (Pall Life Sciences, Victoria, Australia). Other stock solutions were prepared accordingly: 2.5 mg/mL of phytohaemagglutinin (PHA) in RPMI-1640 medium, stored at -20°C; 5 mg/mL of concanvalin-A (Con A) in RPMI-1640 medium, stored at -20°C; 1 mg/mL of phorbol 12-myristate 13-acetate (PMA) in DMSO, stored at -20°C and 5 mg/mL of fluticasone propionate in ethanol, stored at 4°C.

### Desulfation of Enoxaparin Fraction

#### Complete desulfation

A solution containing 8 mg/mL of enoxaparin fraction was subjected to acid hydrolysis for complete removal of sulfate groups as described previously [26]. Briefly, 1 mL of nitric acid was added to the fraction in a capped glass vial and the solution was heated at 80°C overnight before adding another 0.2 mL of hydrogen peroxide. The temperature was raised to 110°C for an additional 6 hours. The mixture was neutralised using 1 M sodium hydroxide and diluted with 4 mL of Milli-Q water. Finally 200 μL of this solution was further diluted to 4mL.

#### N-desulfation

A solution containing 8 mg/mL of enoxaparin fraction was incubated at 50°C for 30 minutes in the presence of tetrahydrofuran (133 μL) and water (7μL) for partial *N*-desulfation as described previously [27] with minor modifications. The mixture was neutralised using 0.1 M sodium hydroxide. The resulting product was evaporated to dryness and precipitated by the addition of anhydrous methanol (80% *v/v*) followed by centrifugation at 3000 rpm for 10 minutes. The supernatant was carefully discarded and samples were kept at 4°C overnight. Any traces of methanol were removed using a miVac DNA centrifugal concentrator and the remaining precipitants were dissolved in 1 mL Milli-Q water.

#### Selective 2-O-/3-O-desulfation

Selective 2-*O*-/3-*O*-desulfation of enoxaparin fraction was performed as described previously [27]. Briefly, the fraction (8 mg/mL) was dissolved in 0.1 M sodium hydroxide (200 μL) and then lyophilised to dryness. The residues were dissolved in Milli-Q water (0.5 mL) and the pH was neutralised by addition of acetic acid. Finally, the resulting mixture was precipitated using anhydrous methanol as described above and dissolved in 4 mL Milli-Q water.

#### Selective 6-O-desulfation

Selective 6-*O*-desulfation of enoxaparin fraction was performed as previously described [27]. Briefly, the fraction (8 mg/mL) was mixed with equal volumes (1mL) of tetrahydrofuran (solvent) and *N*-methyl-*N*-(trimethylsilyl) trifluroacetamide (silylating agent). The mixture was incubated at 50°C for 9 hours, evaporated to dryness and precipitated using anhydrous methanol as described above and finally dissolved in 4 mL Milli-Q water.

Each desulfated fraction was tested for its inhibitory effect on PBMC-induced release of cytokines. After desulfation, free sulfate groups were removed using a 1000Da cut-off filter (Millipore, NSW, Australia) at 15000 rpm for 10 minutes. The concentrated supernatant was dissolved in 1 mL of Milli-Q water for further use.

### PBMC Culture

Cells were cultured in 24-well cell culture plates in complete medium at a concentration of 1 × 10^6^ cells/mL/well. Cells were incubated with 1, 2.5, 5, 7.5, 10, 20 or 40 μg/mL of PHA for 72 hours to identify the optimum concentration for the release of TNF-α. To measure the effect of fractions on the release of cytokines, the cells were stimulated with 10 μg/mL PHA in the presence or absence of IC-derived enoxaparin fractions. The effect of fractions was compared to positive control after addition of 0.5 ng/mL fluticasone in the presence of 10 μg/mL PHA. The target specific effect of enoxaparin fraction on cytokine release was examined after stimulation of cells with either 10 μg/mL Con A or 5 ng/mL PMA. The time dependency of enoxaparin fraction was determined after addition of the fraction at different time points in the presence of 10 μg/mL PHA and vice versa. The effect of various desulfated enoxaparin fractions was investigated in the presence of 10 μg/mL PHA. After 72 hours of incubation (37°C, humidified 5% CO_2_ atmosphere), culture supernatants from each well were collected and analysed for the determination of cytokine concentrations using flow cytometry.

Each cell supernatant containing cell stimulants and/or various enoxaparin fractions, desulfated enoxaparin fractions or fluticasone was prepared and analysed in triplicate.

### Measurement of Cytokine Release

The amounts of IL-4, IL-5, IL-13 and TNF-α secreted into the cell culture supernatant were analysed using a cytometric bead assay (Cytometric bead array flex sets;BD Biosciences, NSW, Australia),which was performed according to the manufacturer’s instructions. Briefly, supernatant from different treatments was incubated with the beads of interest for 3 hours in the dark, prior to washing, resuspending and transferring to 96 well plates. The samples were run on a flow cytometer (FACS Canto, Becton Dickinson, CA, USA) and gates were set to include only singlet populations of beads. The assay detection limit was stated by the manufacturer to be ≈ 3 pg/mL, with recovery rates of ≥ 80% of protein. Data was analysed by FACS Diva Software. Each sample was analysed in triplicate.

### PBMC Viability Assays

The effect of enoxaparin fractionon cell viability after 72 hours of incubation was assessed using two methods, trypan blue dye exclusion assay and lactate dehydrogenase (LDH) release into culture supernatant. Both assays employed routinely used methods. Cytotoxicity of treatment was determined using the LDH *in-vitro* toxicology assay kit (Sigma-Aldrich, NSW, Australia), according to the manufacturer’s instructions. Briefly, PBMC culture supernatants were centrifuged at 250g for 4 minutes. An aliquot containing 50 μL of cleared supernatant was mixed with 100 μL of a solution containing LDH assay substrate, LDH dye and LDH cofactor and incubated at room temperature for 20 minutes before the reaction was terminated by the addition of 15 μL of 1 N hydrochloric acid. Absorbance at 490 nm was measured spectrophotometrically using a plate reader (Spectra Max M2 microplate reader, Sunnyvale, CA). Each sample was measured in triplicate.

### Nuclear Magnetic Resonance (NMR)Analysis

#### Saccharide information of enoxaparin fraction

Samples for NMR analysis were prepared in 50mM potassium phosphate buffer (KPO_4_) and 99.9% D_2_O. All experiments were carried out on a Bruker Avance III HD 600 MHz spectrometer using a TCI triple-resonance cryogenically cooled probe with z-gradients all controlled by the software Topspin 3.2 (Bruker Corporation, MA, USA). Spectra were recorded at 25°C. Characterisation of fraction was performed at 100 μM using 1D and 2D ^1^H spectroscopy (TOCSY 120 ms, COSY, ROESY 500 ms) and 2D ^13^C-^1^H spectroscopy (HSQC, HSQCTOCSY 120ms) with standard Bruker pulseprograms.

### Putative Binding of Enoxaparin Fraction to PHA

Saturation Transfer Difference-nuclear magnetic resonance spectroscopy (STD-NMR) was used to assess the potential binding of enoxaparin fraction to PHA by using the stddiffgp19.3 pulseprogram from the Bruker library, incorporating suppression of the residual water resonance with a Watergate sequence. The method was validated using a sample of bovine serum albumin (10 μM), tryptophan and glucose (100 μM) following previously published guidelines [28]. STD build-up curves were observed with saturation times of 0.5, 1.0, 2.0 and 5.0 seconds, respectively, with a range of shaped pulse power levels from 30–60 dB. An optimum saturation of 2 seconds was chosen from the steeper portion of the build-up curve, and 32 dB of saturation pulse power with an on-resonance excitation pulse at -1 ppm and off resonance at 30 ppm. 128 transients were recorded in 8192 datapoints with a relaxation delay of 1.5seconds. The interleaved spectra were processed and difference spectrum calculated using Topspin 3.2 incorporating the stdsplit macro function. The potential binding of enoxaparin fraction to PHA was examined using a 500 μL sample of fraction (100 μM) and PHA (5 μM) prepared in D_2_O buffered with 50 mM KPO_4_ at pH 7.0. The above experimental design was repeated over a range of saturation times from 0.5-5seconds at 32 dB saturation pulsepower with up to 2000 transients recorded.

### Statistical Analysis

Results are presented as mean ± standard deviation (SD) or as percentage change in the release of cytokines following different types of treatments, compared either to PHA, Con A or PMA only controls. Each donor blood sample was treated as control (cells + cell stimulant) as well as treatment (cells + cell stimulant + enoxaparin fractions). The statistical analysis was performed on the raw data using a total mean response from all the mean values of controls as well as treatments. Statistical analysis was performed using GraphPad Prism (version 6, GraphPad Software Inc, CA, USA). Given the fewer number of observations, statistical significance were evaluated using non-parametric Wilcoxon-signed ranked test and Kruskal-Wallis test, where applicable. Mann-Whitney U tests were then utilised to compare inter-group differences in place of Dunnett’s post-hoc analysis. A *p*-value of <0.05 was considered statistically significant.

## Results

### Cytokine Release

**Fig 1:**
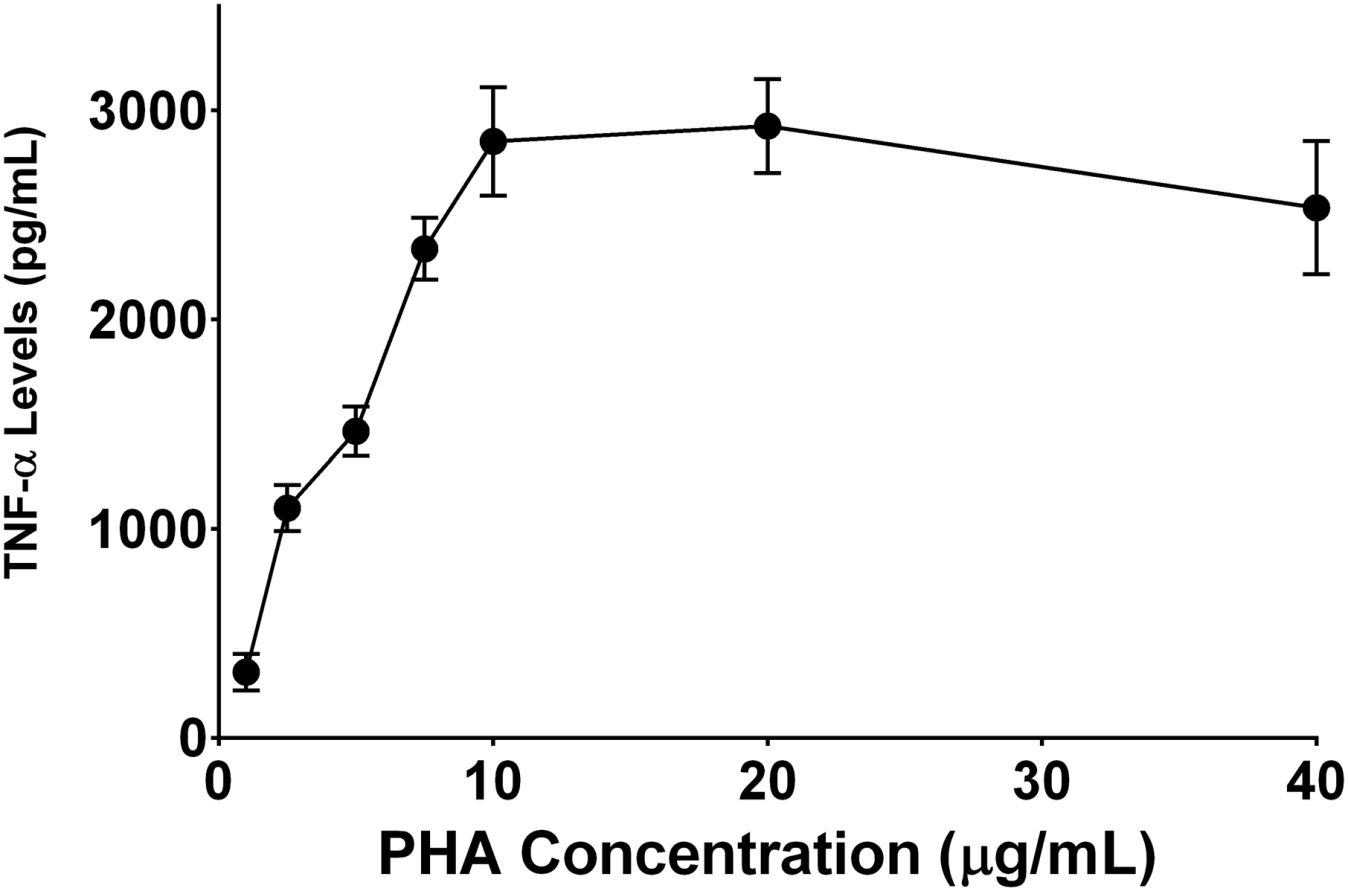
Concentration-dependent effect of PHAon the release of TNF-α. PBMCs from allergic asthmatic subjects (n = 2) were stimulated with various concentrations of PHA (1 to 40 μg/mL) for 72 hours. Data is presented as mean ± SD.

**Fig 2:**
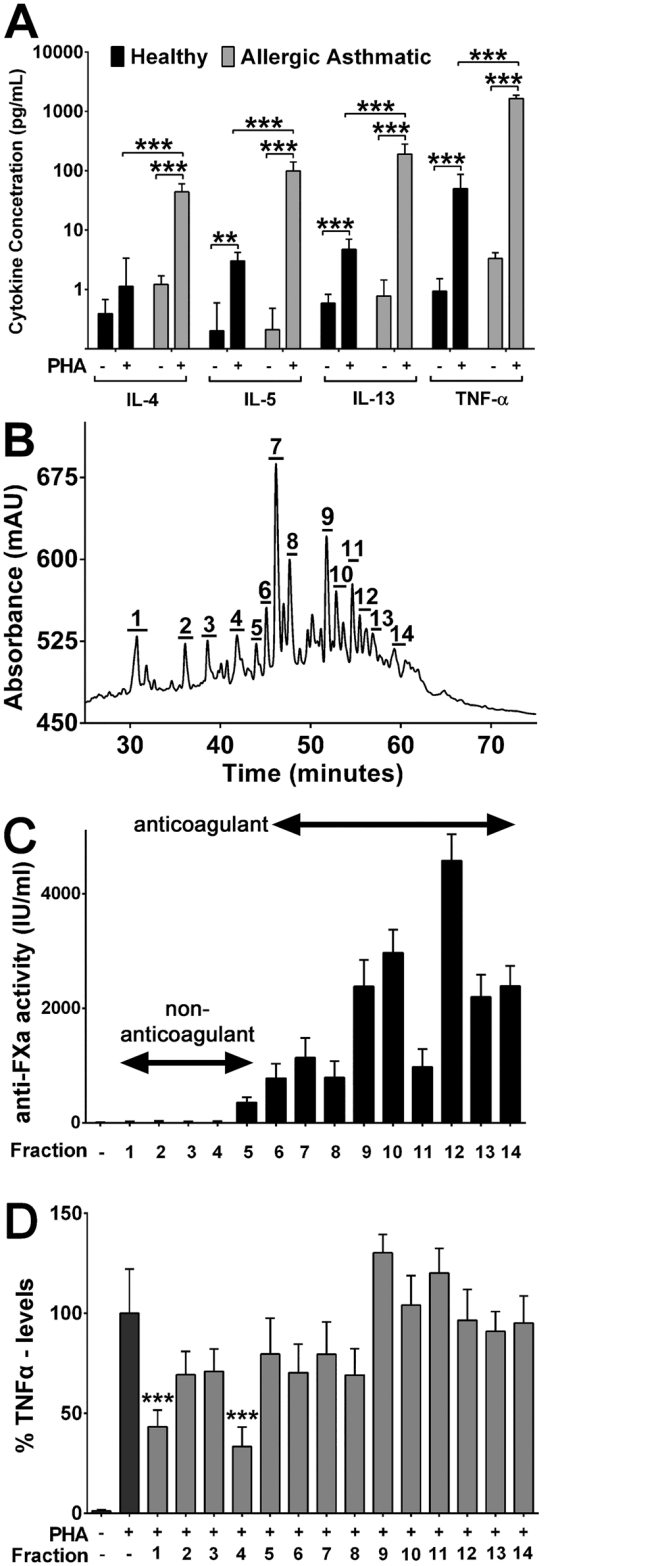
Effect of enoxaparin fractions on *ex-vivo* cytokine release. **(A)** PHA-induced release of cytokines (logarithmic scale) from PBMCs of healthy (n = 5) and allergic asthmatic (n = 10) subjects. Each sample was analysed in triplicate and the data is presented as the average of five and ten individual samples for healthy and allergic asthmatic subjects, respectively. Data is presented as mean ± SD. **(B)** Ion-chromatographic (IC) separation of enoxaparin, fractionated using a CarboPac PA100 semi-preparative column; a 32–74% NaCl gradient over 70 minutes; 2 mL/minute flow rate and a detection wavelength of 232 nm. The numbers indicate the area of all the fractions collected. Data represents a typical experiment out of five independent experiments. **(C)** Anticoagulant activity of IC-separated enoxaparin fractions. Data is presented as mean ± SD (n = 3). Anticoagulant activity was observed from fraction 6 onwards.**(D)** Inhibition of TNF-α release by different fractions of enoxaparin. TNF-α was released by PBMCs of allergic asthmatic subjects (n = 10) after *ex-vivo* stimulation with PHA. The relative percentile amount of each fraction (fraction 1 to 14) present in 500 μg/mL of intact enoxaparin was: 9.18%, 4.24%, 6.12%, 5.15%, 2.99%, 4.78%, 20.32%, 11.4%, 10.53%, 7.35%, 5.98%, 4.6%, 2.95% and 5.06% respectively. Data is presented as mean ± SD. ***p<0.001 versus the PHA-stimulated control.

The release of TNF-α from PBMCs of allergic asthmatics in the presence of different concentrations of PHA is shown in Fig 1. Concentration-dependent increase in the release of TNF-α was observed. The release of TNF-α was found to be 315.1, 1099.0, 1465.8, 2337.8, 2852.7, 2925.4 and 2534.6 pg/mL at 1, 2.5, 5, 7.5, 10, 20 and 40 μg/mL respectively. The concentration of PHA at 10 or 20 μg/mL was found to be the most effective in releasing TNF-α from allergic asthmatic subjects. There was no statistical difference in the release of tested cytokine when 10 or 20 μg/mL of PHA was used. Therefore 10 μg/ml of PHA was used for subsequent experiments. The reduction of TNF-αlevels at higher concentrations (40 μg/mL) is most likely due PHA-induced cell apoptosis which has been reported previously [29,30]. We selected 72 hours as the incubation time period for all the experiments as previous studieshas shown that 10 μg/mL of PHA is optimum to give an appropriate response for the cytokines of interest in 3-day cultures [31]. After 72 hour incubation with PHA, PBMCs from allergic asthmatic subjects released significantly higher levels of IL-4, IL-5, IL-13 and TNF-α than PBMCs of healthy controls (Fig 2A) with TNF-α showing the highest levels of 1652.6 pg/mL overall. The highest proportional change was observed for IL-5 in PBMCs from allergic asthmatic individuals, where its levelswere increased by 98.7%.

### Separation of Enoxaparin

Intact enoxaparin has shown to inhibit the release of IL-4, IL-5, IL-13 and TNF-α [4]. However, the potential use of enoxaparin in various inflammatory disorders is largely hindered by the risk of bleeding caused by the presence of anticoagulant fractions [32,33]. To determine whether the observed activity of intact LMWH is independent to its anticoagulant effect, enoxaparin was separated into different fractions with or without anticoagulant activity (Fig 2B and 2C). The approximate saccharide composition of each fraction ranging from two saccharide units (dp2; molecular weight ~600 Da) to 24 saccharides (dp24; molecular weight ~8000 Da) was previously determined using size-exclusion chromatography [24]. Each IC-derived enoxaparin fraction was collected, desalted and quantified. The concentration of NaCl in each desalted fraction of enoxaparin was found to be less <0.9% (*w/v*). This NaCl content was deemed acceptable since it is known that “normal” saline (0.9%, *w/v*, NaCl) is isotonic with body fluids and is used to dilute the fractions while performing the anti-factor Xa analysis and to wash the cultured cells during the determination of anti-inflammatory activity. Each fraction was tested for their effect on cytokine release from stimulated PBMCs of allergic asthmatic subjects.

### Effect of IC-derived Enoxaparin Fractions on Cytokine Release

Since PHA induced the highest levels of TNF-α in the culture medium, this cytokine response was initially selected to test the effect of enoxaparin fractions in this cellular system. The isolated enoxaparin fractions modulated TNF-α release to different extents (Fig 2D). While the majority of fractions did not significantly change the levels of TNF-α, fractions 1 (disaccharide) and 4 (tetrasaccharide) significantly inhibited TNF-α release by 57.6 and 67.5% respectively. Interestingly, for the other two fractions (9 and 11) a trend towards increased TNF-α release was detected, which did not reach significance. To investigate the anti-inflammatory activity of enoxaparin in more detail we selected fractions 1and 4 for further analysis.

### Effect of Fractions 1 and 4 on Cytokine Release

**Fig 3:**
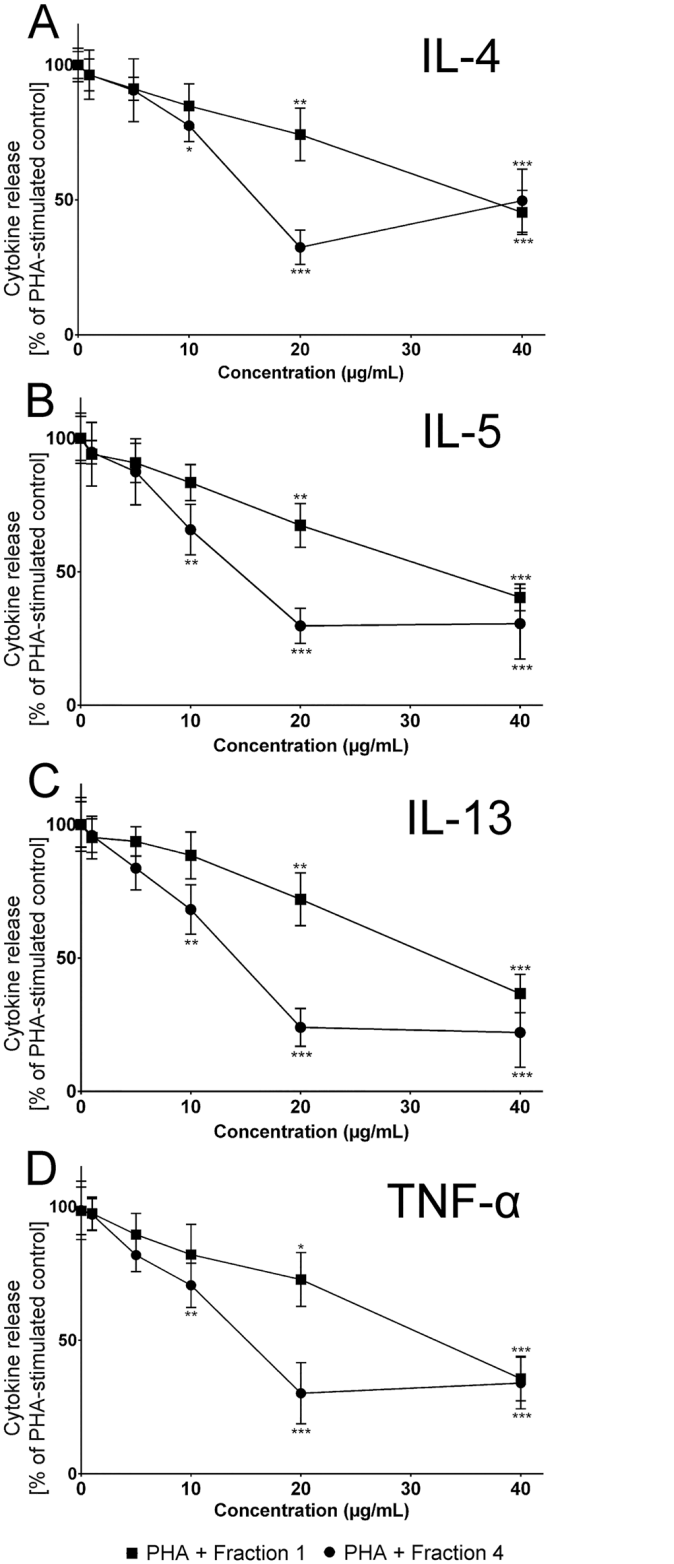
Concentration-dependent suppression of cytokine release by enoxaparin fractions. Inhibition of cytokine release by 0 to 40 μg/ml of enoxaparin fractions 1 and 4. Cytokines [IL-4 **(A)**, IL-5 **(B)**, IL-13 **(C)** and TNF- α **(D)**] were released by PBMCs of allergic asthmatic subjects (n = 5) after *ex-vivo* stimulation with PHA. Data is presented as percentage of PHA-stimulated control. *p<0.05, **p<0.01 and ***p<0.001 versus PHA-stimulated control.

Using PBMCs from asthmatic subjects, the concentration dependent effects of fractions1 and 4 were tested with regards to their inhibitory activity towards the release of IL-4, IL-5, IL-13 and TNF-α (Fig 3A–3D). Both fractions significantly inhibited the release of all cytokines in a concentration-dependent manner. While fraction 1 showed maximal inhibition of cytokine release at 40 μg/mL, fraction 4 maximally reduced cytokine release at 20 μg/mL. Higher concentrations of fraction 4 did not result in a stronger inhibition. Instead a plateau of about 24–49% residual cytokine release compared to the PHA-stimulated control appeared to be the maximal possible level of inhibition by these fractions. Overall, inhibition byfraction 4 was greater than fraction 1, with maximum inhibition by 68.6%, 70.2%, 76.0% and 69.8% for IL-4, IL-5, IL-13 and TNF-α, respectively. The inhibitory effect of fraction 4 on TNF-α release in the presence of 20 μg/mL of PHA was also investigated and the percentage inhibition was found to be 67.14% which was not statistically significant to the observed inhibition seen with 10 μg/mL of PHA. The observed inhibitionof cytokine release by the two enoxaparin fractions 1 and 4 was comparable to the corticosteroid, fluticasone, one of the currently used agentsin the management of asthma (S1 Fig). There is evidence that the anti-inflammatory responses of heparins are known to be dependent on their chain lengths as well as sulfation pattern. For instance, it has been shown that the anti-proliferative activity exhibited by heparin requires oligosaccharide chains greater than six saccharides [34]. Moreover, *O*-sulfated heparin retains anti-proliferative activity and oversulfation at *O*-positions enhances this activity [35,36]. Similarly, the observed differences in the inhibition of tested cytokines by different fractions could possibly be due to various degree of polymerisation, where a minimum of two and a maximum of four saccharide units are required to bind cellular receptors and exhibit inhibitory effects. The difference in the observed inhibitory effects of fraction 1 and fraction 4 could be high degree of sulfonation especially at *O*-positions in fraction 4 resulting in enhanced inhibition of cytokine release.

### Effect of Fraction 4 on Cellular Viability and Proliferation

**Fig 4:**
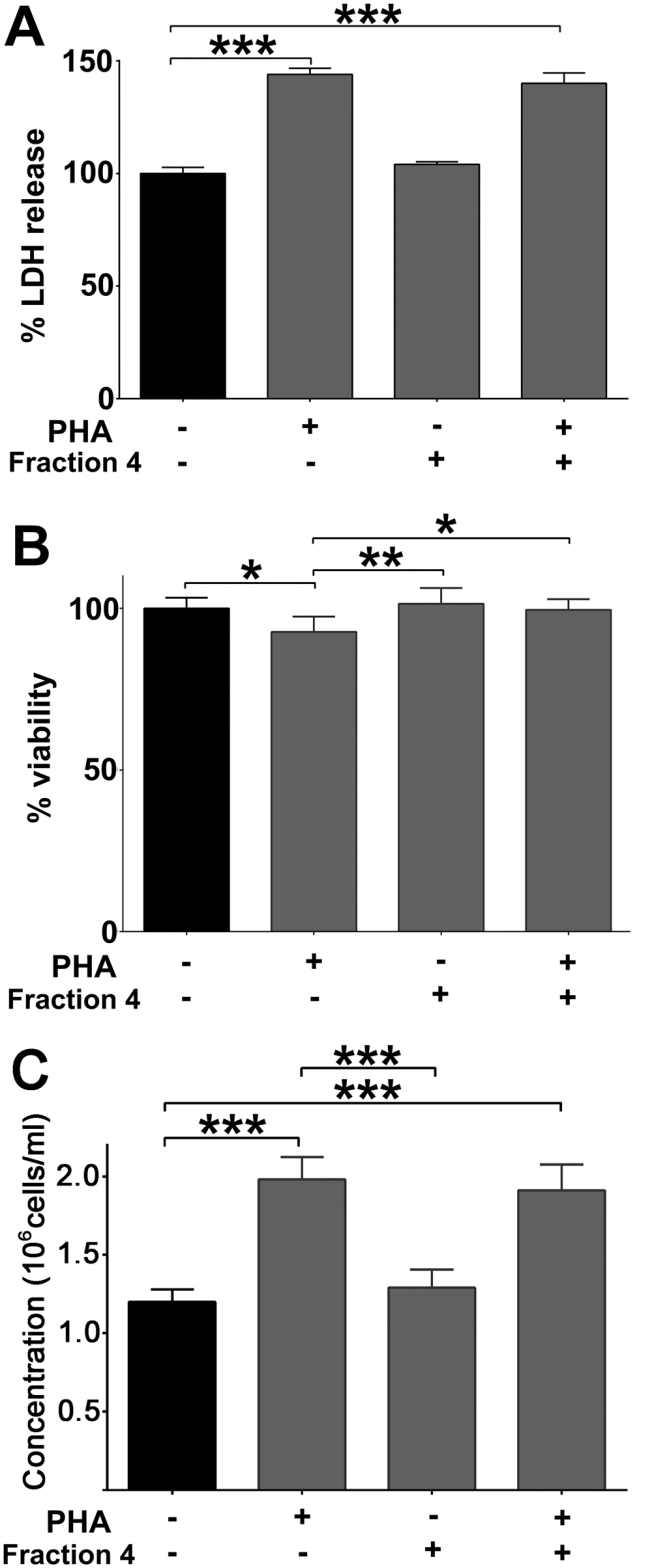
Effect of fraction 4 on cellular viability. **(A)** Viability of PBMCs is presented as mean % LDH release ± SD from allergic asthmatic subjects (n = 3). Cells were incubated for 72 hours in the presence of PHA, fraction 4 or PHA + fraction 4.*p<0.05 and **p<0.01 versus unstimulated control. **(B)** Viability of PBMCs from allergic asthmatic subjects (n = 3) was determined by trypan blue dye exclusion test and is presented as % of viable cells remaining after 72 hours of incubation with PHA, PHA + fraction 4 or fraction 4 alone. Data is presented as mean ± SD. **(C)** Proliferation of PBMCs from allergic asthmatic subjects (n = 3) was determined after 72 hours in the presence of PHA, fraction 4 alone or PHA + fraction 4. Cells were counted after incubation for 72 hours and expressed as million cells/mL. Data is presented as mean ± SD.

To rule out that the observed inhibition of PBMC activation by fraction 4 arose as a consequence of cytotoxic effects or changes in cellular proliferation, we assessed cell viability in the presence or absence of PHA and/or fraction 4 by detecting LDH release into the cell culture supernatant (Fig 4A). Fraction 4 did not induce any increase of extracellular LDH while PHA, as expected, showed significant toxicity. This toxicity was not affected by co-incubation of PHA with fraction 4 (Fig 4A). Consistent with the LDH-release results, no signs of cytotoxicity were detected in the presence of fraction 4 alone when using trypan blue exclusion as the toxicity readout (Fig 4B). In this case, only mild signs of toxicity by PHA could be detected, which when combined with fraction 4 was no longer significantly different to unstimulated PBMCs. Based on cell counts, PHA was found to induce significant proliferation of PBMCs. Fraction 4, on the other hand, showed no effect on cell proliferation when compared to either PHA or untreated control (Fig 4C).

### Time and Target Specificity of Fraction 4

**Fig 5:**
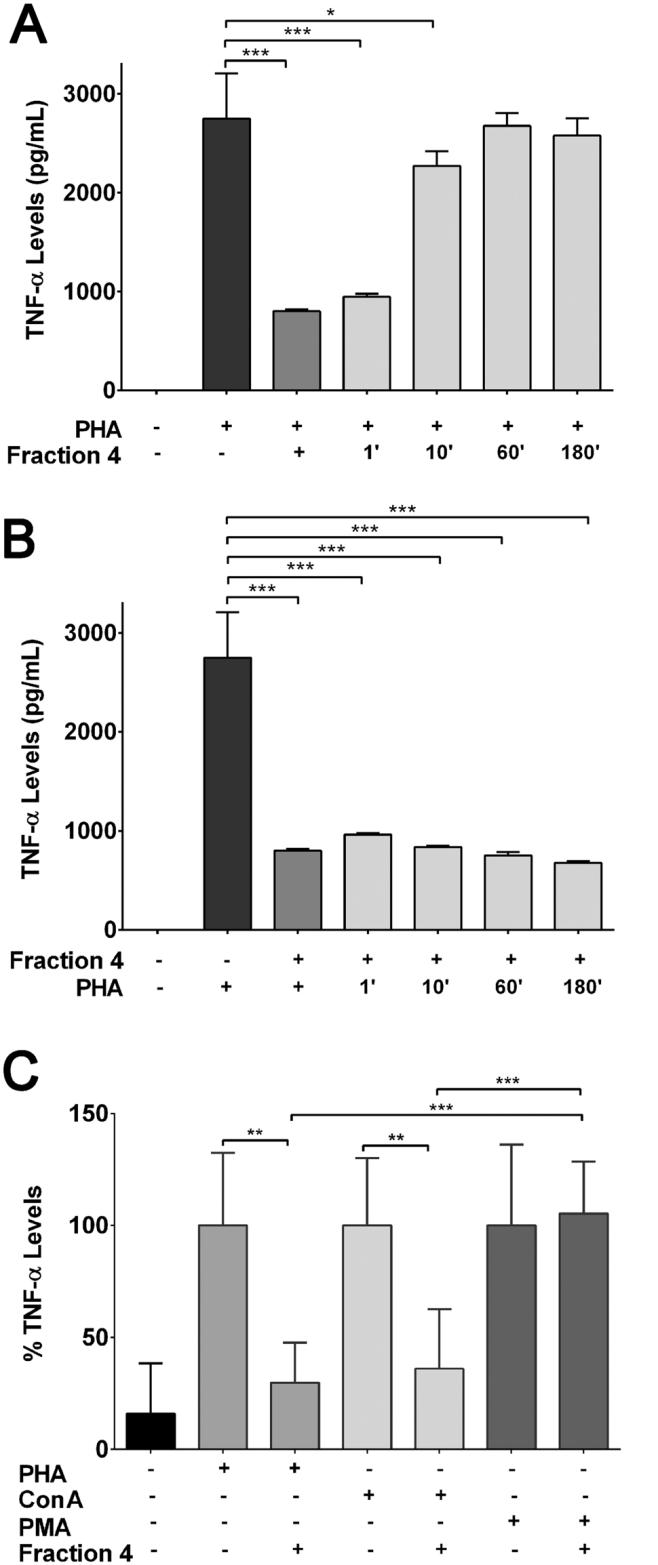
Time- and target-dependent inhibitory effect of fraction 4. Effect of fraction 4 on TNF-α release by activated PBMCs of allergic asthmatic subjects (n = 3). **(A)** Addition of fraction 4 at different time points after the addition of PHA and **(B)** addition of PHA at different time points after the addition of fraction 4. Data is presented as mean ± SD. *p<0.05 and ***p<0.001 versus PHA only control. **(C)** Effect of fraction 4 on TNF-α release from activated PBMCs of allergic asthmatic subjects (n = 3) after stimulation with either PHA (10 μg/mL), Con A (10 μg/mL) or PMA (5 ng/mL). Data is presented as mean ± SD. *p<0.05, **p<0.01 and ***p<0.001 versus PHA or Con A or PMA only control respectively.

To gain a better understanding of the inhibitory mechanism of the fraction 4 of enoxaparin we investigated the time dependency of this process. When the PBMCs were treated with fraction 4 at different times after PHA stimulation, the inhibitory effect was lost after 10 minutes (Fig 5A). Maximal inhibition of TNF-α release was only observed if fraction 4 was added concurrently with PHA or at most 1 minute after PHA stimulation (Fig 5A). On the other hand, when PBMCs were pre-treated with fraction 4 up to 180 min before PHA stimulation, all time points showed effective inhibition of TNF-α release (Fig 5B).

To understand if this inhibitory effect of fraction 4 was restricted to a specific form of stimulation or molecular target, three different modes of activation were tested (Fig 5C). Consistent with prior results, fraction 4 significantly inhibited the release of TNF-α after stimulation of PBMCs with PHA. Similarly, a significant inhibition of TNF-α release was also observed after stimulation of PBMCs with ConA in the presence of fraction 4 (Fig 5C). Interestingly, the inhibitory effect of fraction 4 on TNF-α release was found to be completely absent when PMA was used to stimulate the PBMCs (Fig 5C).

### NMR Analysis

#### Saccharide information of fraction 4

NMR analysis was performed to identify the number of saccharide units present in fraction 4 of enoxaparin. A 2D ^13^C-^1^H multiplicity edited HSQC of fraction 4 specified the presence of four sugar units with single set of signals detected for each of the four sugar units (S2 Fig). Therefore, fraction 4 of enoxaparin was confirmed to have a tetrasaccharide sequence.

### Lack of PHA Binding to Fraction 4

PHA is known to bind complex polysaccharides as well as the T-cell receptor [37]. Therefore, using STD-NMR we examined if fraction 4 of enoxaparin could bind to PHA directly to rule out an experimental artefact. Since direct binding of the two molecules could impair the ability of PHA to bind and activate the T-cell receptor we needed to confirm that the observed inhibition of T-cell activation by fraction 4 was not due to this potential *in-vitro* artefact of our experimental cell system. In the absence of any available small-molecule as a putative PHA binder, the STD-NMR experiments were validated using bovine serum albumin, tryptophan and glucose as described previously. As anticipated, a clear saturation as seen in spectrum B, reveals binding of tryptophan to bovine serum albumin, while in the same spectrum, no saturation of the glucose component confirmed its lack of binding (S3 Fig). Under identical experimental conditions, PHA did not bind fraction 4 since no saturation of this fraction was detected after excitation (S4 Fig). Spectrum A shows fraction 4 resonances from the ^1^H-noespr1d experiment and spectrum B shows the only visible signals from excitation of PHA (S4 Fig).

### Effect of Desulfated Fraction 4 on Cytokine Release

**Fig 6:**
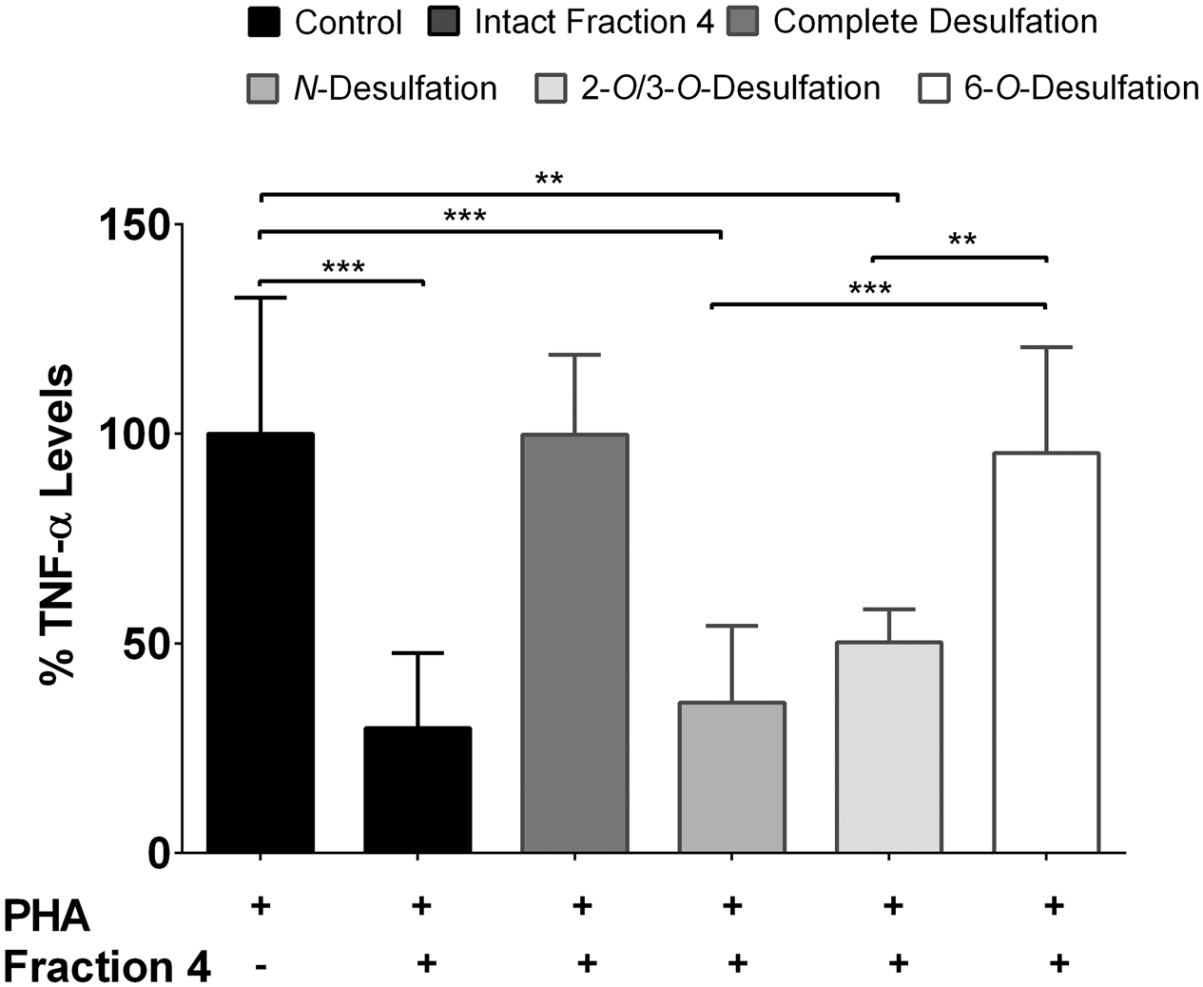
Effect of desulfation on anti-inflammatory activity of fraction 4. PBMCs of allergic asthmatic subjects (n = 3) were stimulated with PHA in the presence of either completely desulfated, *N*-desulfated, 2-*O*/3-*O*-desulfated or 6-*O*-desulfated fraction 4 of enoxaparin. Data is presented as mean ± SD. *p<0.05, **p<0.01 and ***p<0.001.

Since enoxaparin oligosaccharides are enriched with sulfate groups that are thought to be important for its anti-inflammatory activity, we examined the influence of sulfate groups by testing fraction 4 after desulfation of specific moieties with regards to their influence on the suppression of TNF-α release (Fig 6). It was observed that the inhibitory effect of fraction 4 was significantly reduced after complete and 6-*O*-desulfation of the fraction while *N*- and 2-*O*/3-*O*-desulfated fraction had no significant influence on the release of TNF-α. The inhibitory effect on the release of TNF-α after 6-*O*-desulfation was found to be reduced by more than 95%.

## Discussion

Beyond their well-established effect on blood-coagulation, LMWHs have been recognised for many other biological activities, including anti-inflammatory activity. Substantial evidence demonstrates that LMWHs could be effective for managing many inflammatory disorders including, asthma [38], ulcerative colitis [39] and lichen planus [40]. Although studies investigating the anti-inflammatory potential of different LMWHs have shown varying results, overall a clear benefit has been demonstrated, with enoxaparin being one of the most active LMWH [18,41,42].

**Fig 7:**
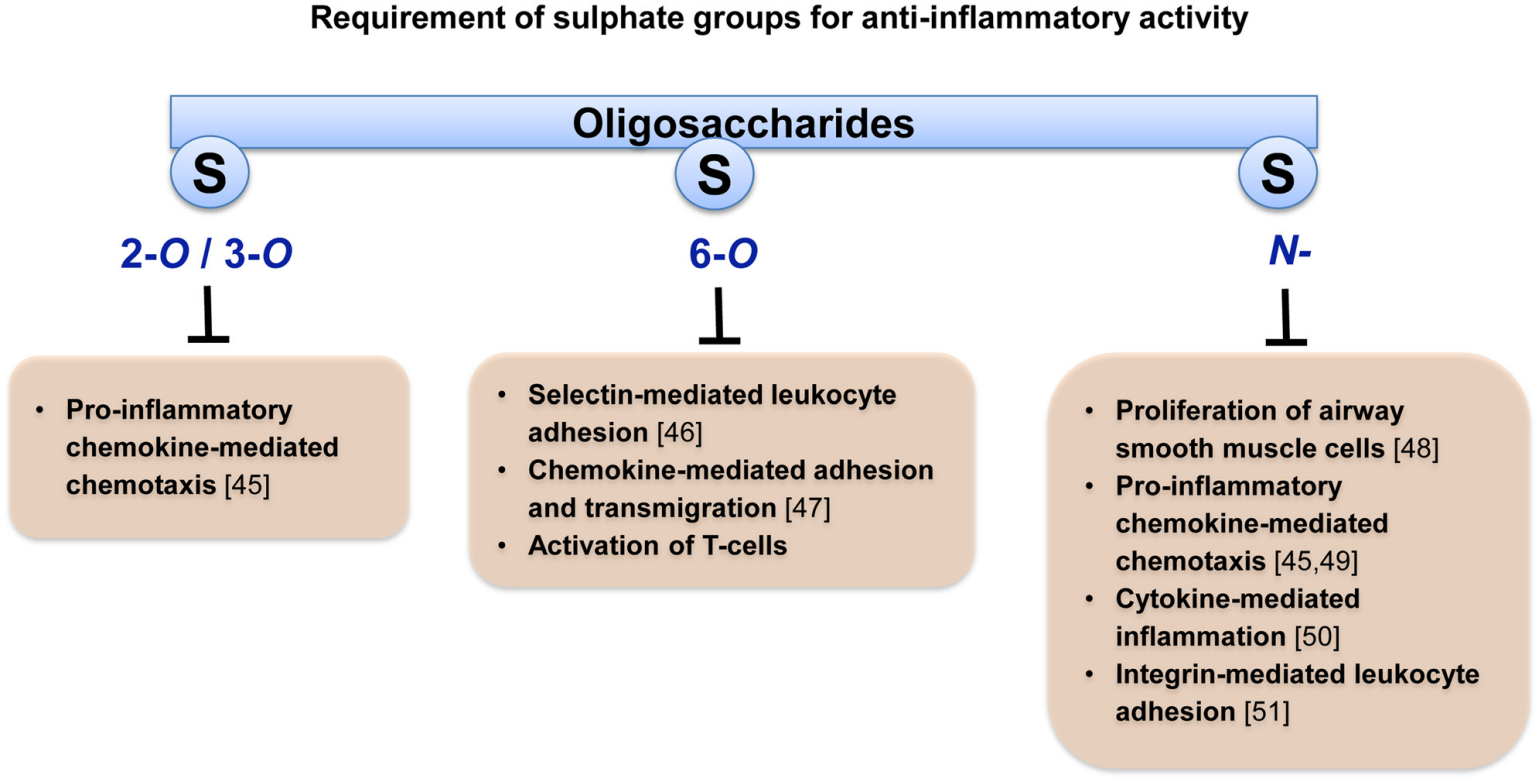
Schematic representation of the different sulfate groups required to elicit various anti-inflammatory responses.

Nevertheless, the use of these complex macromolecules in inflammatory disorders is unlikely to progress because they harbour the inherent risk of bleeding complications due to their anticoagulant activities. To avoid suchrisk associated with the intact LMWHs, a range of attempts have been made to separate non-anticoagulant and anticoagulant fractions of oligosaccharides. One available approach is to chemically or enzymatically depolymerise the parent LMWH. However, depolymerisation can result in a structural modification of oligosaccharides, with a loss of biological activities exhibited by the parent molecules [43,44]. For example, some oligosaccharides of LMWHs are heat-sensitive and can undergo chemical modification, especially removal of important sulfate groups, during the elevated temperatures of the depolymerisation process [25]. Furthermore, the presence and location of specific sulfate groups is critical to elicit anti-inflammatory effects (Fig 7.) [45–51]. For instance, sulfate groups at the 6-*O* position are essential for inhibiting leukocyte adhesion while sulfate groups at the 2-*O*/3-*O* and *N*-positions are required for the inhibition of chemotaxis or proliferation [45,46,48]. Therefore, the loss of any of these sulfate groups as a result of depolymerisation will impair a specific aspect of the anti-inflammatory response exhibited by the parent LMWH. Consequently, the protocol for separation, isolation and purification of oligosaccharide fractions from intact LMWH, as described here, represents a major step towards the identification of a sub-fraction that retains full anti-inflammatory activity of the parent molecule [4]. Our results demonstrate for the first time the requirement of 6-*O* sulfate groups for the suppression of PBMC activation while, removal of 2-*O*/3-*O-* and *N*-sulfate groups had little effect. Although selective desulfation procedures were performed using well established and validated analytical methods, a major limitation of the current study is that we did not investigate and confirmed the integrity and purity of the desulfated fraction of enoxaparin.

Among the potential mechanisms that could account for the anti-inflammatory effect of heparins, their interaction with a broad range of bioactive molecules involved in the process of inflammation, such as cytokines, growth factors, adhesion molecules, tissue-destructive enzymes and cytotoxic mediators has been proposed [52]. However, the mode(s) of action behind the observed anti-inflammatory effect of various types of heparins in disorders like asthma is poorly understood. It was previously suggested that the anti-inflammatory activity of heparins could be dependent on the inactivation of nuclear factor-kappa B, the inhibition of 1,4,5-inositol triphosphate-induced signalling or interaction with the CD11b receptor [18,20,53]. Here we describe for the first time, another mechanism by which enoxaparin fractions can inhibit a PBMC-mediated inflammatory response induced by receptor-activating ligands, such as PHA or ConA [54,55]. Since the lectins, PHA and ConA are known to bind the CD3- and CD28-T cell receptor complex, as well as polysaccharides, the anti-inflammatory effects we observed by enoxaparin fractions could be a result of either: i) saturating the polysaccharide binding site of PHA and thereby blocking its binding to the T-cell receptor; ii) binding and blocking the PHA-binding site on the T-cell receptor; or iii) binding to an allosteric binding site on the T-cell receptor which prevents T-cell receptor activation. Since the NMR analysis demonstrated that the enoxaparin fraction 4 does not bind to PHA, a specific or allosteric binding to cellular receptor(s) complex is likely. This hypothesis is supported by our observation that the enoxaparin fraction 4 was unable to prevent the inflammatory response when PBMCs were stimulated with PMA, which activates protein kinase C (PKC) downstream of the T-cell receptor [56]. Finally, the general binding between cell surface T-cell receptors and PHA or ConA occurs rapidly after co-incubation [57]. For maximum activation and subsequent proliferation it has been shown that the antigen receptor has to be engaged for at least 2 hours [57,58] but a variety of immediate early genes such as interferon-gamma are induced after 30 minutes [59]. Based on the time-dependent inhibition of T-cell activation observed, our results strongly suggest that the potential mechanism by which enoxaparin fractions suppress the inflammatory response is by directly interacting with cell surface receptors and is covering different signalling pathways since both interleukins and TNF-α are supressed. It remains to be seen if this suppression is limited to activation through plant lectins or is extended to antigen-specific activation of the T cell receptor. The identification of specific unmodified enoxaparin fractions paves the way to map the specific binding site(s) involved in future studies.

In summary, the two identified fractions responsible for the anti-inflammatory effect of enoxaparin are composed of two and four saccharides. Therefore, the structure of these fractions eliminate the potential risk of bleeding as a minimum chain length of five saccharide residues (pentasaccharide) is required for the anticoagulant effect. Given the wide range of inflammatory mediators involved in the pathogenesis of asthma, supressing the release of multiple cytokines, as seen with the identified fractions, could provide significant clinical benefits. However, using more specific cellular models such as purified T-cells, anti-CD3/CD28 or intracellular staining of T cells, further work is required to determine whether enoxaparin fractions: i) act directly on T cell receptors and thereby, inhibit the release of T cell mediated cytokines and ii) retains the anti-inflammatory effect when an allergen other than PHA or Con A is used as a stimuli for the activation of T cells. The preliminary results obtained in the current *ex-vivo* study need to be confirmed by using pre-clinical *in-vivo* studies to determine dose-response relationships and to evaluate different formulation strategies. Due to their hydrophilicity and high molecular weight, intestinal absorption is likely to be very low. However, with respect to their use in asthma, a formulation for inhalation would not only deliver the fraction directly and selectively to its site of action, but it would also avoid systemic exposure to the drug, which overall makes this route of administration highly preferable.

## Supporting Information

S1 Fig Comparison between enoxaparin fractions and fluticasone. Inhibition of cytokine release in the presence of fraction 1 (40 μg/mL), fraction 4 (20 μg/mL) or fluticasone (0.5 ng/mL). Cytokines (IL-4, IL-5, IL-13 and TNF- α) were released by PBMCs of allergic asthmatic subjects (n = 10) after stimulation with PHA (10 μg/mL). Data is presented as percentage of PHA only control. *p<0.05, **p<0.01 and ***p<0.001 versus PHA only control.
(TIF)

S2 Fig Saccharide Information of fraction 4. The 2D ^13^C-^1^H multiplicity edited HSQC spectrum for fraction 4. The blue contours represent signals from carbons with 1 or 3 attached protons and the cyan contours represent carbons with two attached protons, i.e. the CH_2_ moieties of the two glucosamine units. This represents the presence of four sugar units with single sets of signals detected for each of the four sugar units and therefore, fraction 4 of enoxaparin was confirmed to have a tetrasaccharide sequence.
(TIF)

S3 Fig Control data for Saturation Transfer Difference (STD) spectroscopy. Two ^1^H-1D spectra are presented for a solution of bovine serum albumin, L-Tryptophan (Trp) and Glucose (Glc). Spectrum **(A)** is the reference spectrum with attenuation of residual solvent using pre-saturation at the solvent frequency. Spectrum **(B)** is the STD spectrum calculated from the difference of two spectra with excitation off-resonance (30 ppm) and on resonance (-1 ppm). The positive binding of Trp to BSA is indicated by the presence of signals that have obtained their excitation via the protein lattice. Glc does not bind and so does not register in the difference spectrum.
(TIF)

S4 Fig NMR analysis elucidating lack of binding of fraction 4 to PHA. Two ^1^H-1D spectra are shown for the putative binding of fraction 4 toPHA. Spectrum **(A)** is the reference spectrum of fraction 4 and PHA obtained with suppression of residual solvent signal by pre-saturation at the solvent frequency. Spectrum **(B)** is the STD difference spectrum calculated from spectra with an on-resonance pulse at -1 ppm and off-resonance pulse at 30 ppm. Only the baseline of PHA signals can be observed in this trace. No binding of fraction 4 can be detected.
(TIF)
