# Supplementary figures and images for: Non-Anticoagulant Fractions of Enoxaparin Suppress Inflammatory Cytokine Release from Peripheral Blood Mononuclear Cells of Allergic Asthmatic Individuals
Source: PLoS One. 2015 Jun 5;10(6):e0128803. doi: 10.1371/journal.pone.0128803 (PMC4457428; doi:10.1371/journal.pone.0128803)

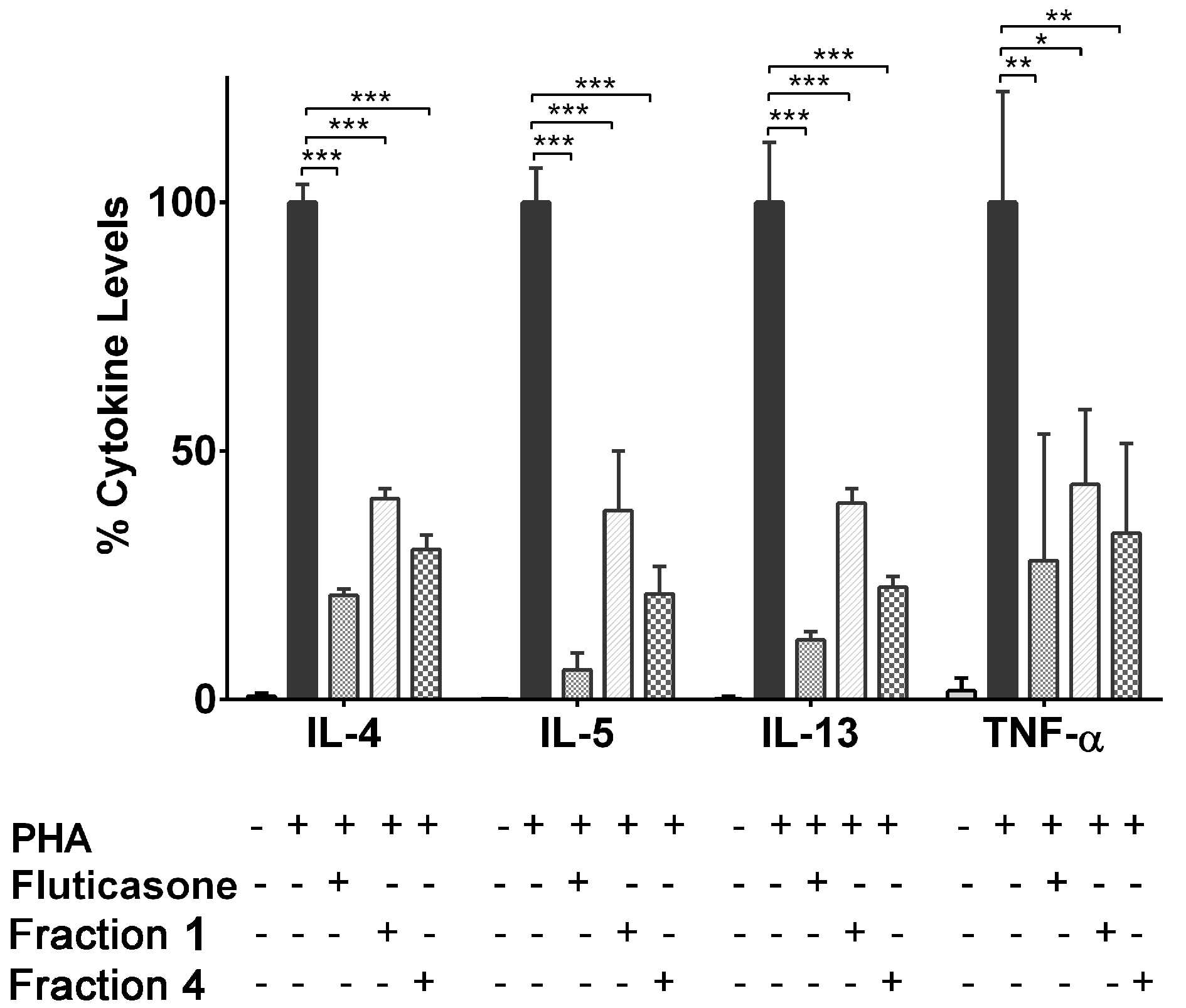

Supplement: S1 Fig — Inhibition of cytokine release in the presence of fraction 1 (40 μg/mL), fraction 4 (20 μg/mL) or fluticasone (0.5 ng/mL). Cytokines (IL-4, IL-5, IL-13 and TNF- α) were released by PBMCs of allergic asthmatic subjects (n = 10) after stimulation with PHA (10 μg/mL). Data is presented as percentage of PHA only control. *p<0.05, **p<0.01 and ***p<0.001 versus PHA only control. (TIF) [file pone.0128803.s001.tif]

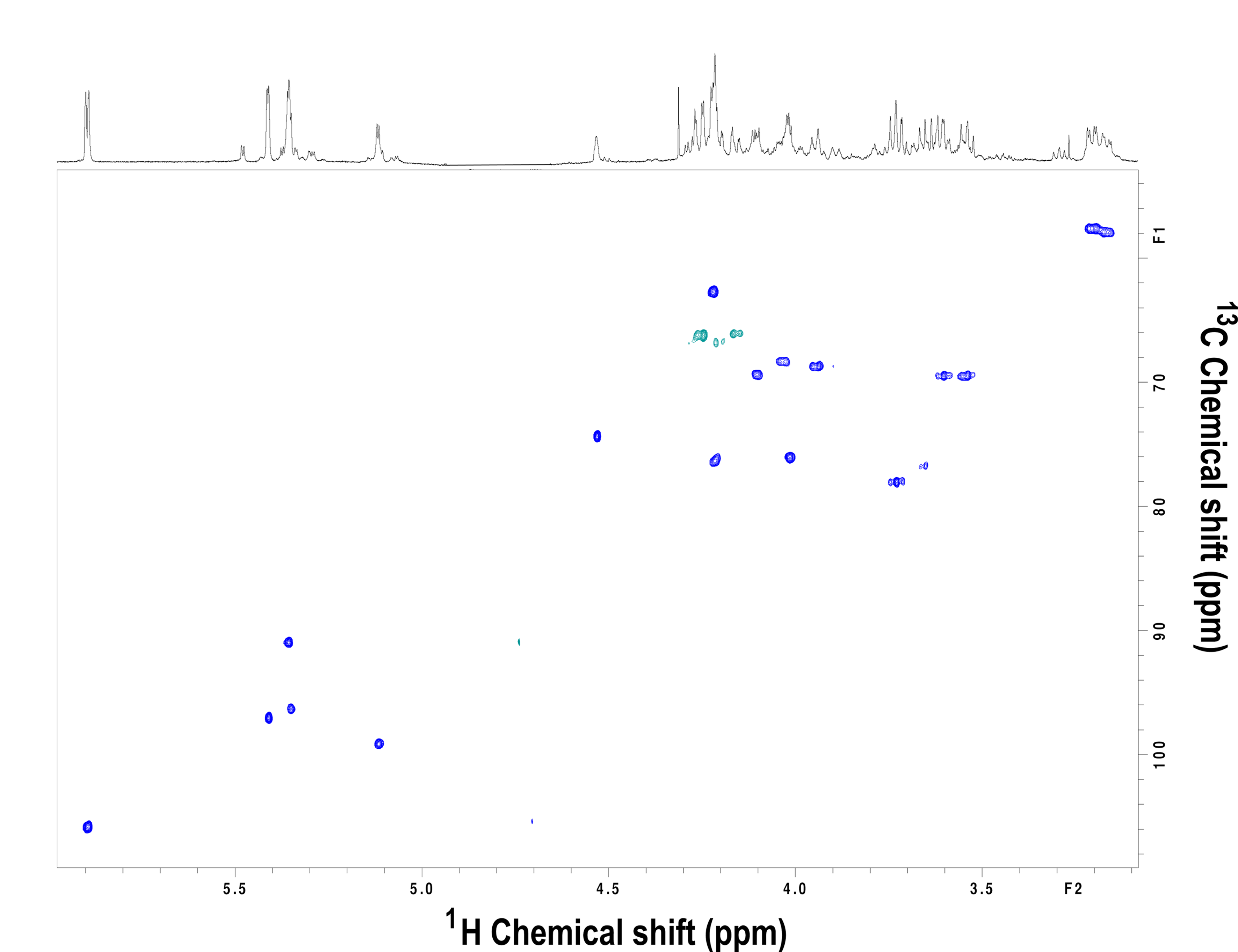

Supplement: S2 Fig — The 2D 13C-1H multiplicity edited HSQC spectrum for fraction 4. The blue contours represent signals from carbons with 1 or 3 attached protons and the cyan contours represent carbons with two attached protons, i.e. the CH2 moieties of the two glucosamine units. This represents the presence of four sugar units with single sets of signals detected for each of the four sugar units and therefore, fraction 4 of enoxaparin was confirmed to have a tetrasaccharide sequence. (TIF) [file pone.0128803.s002.tif]

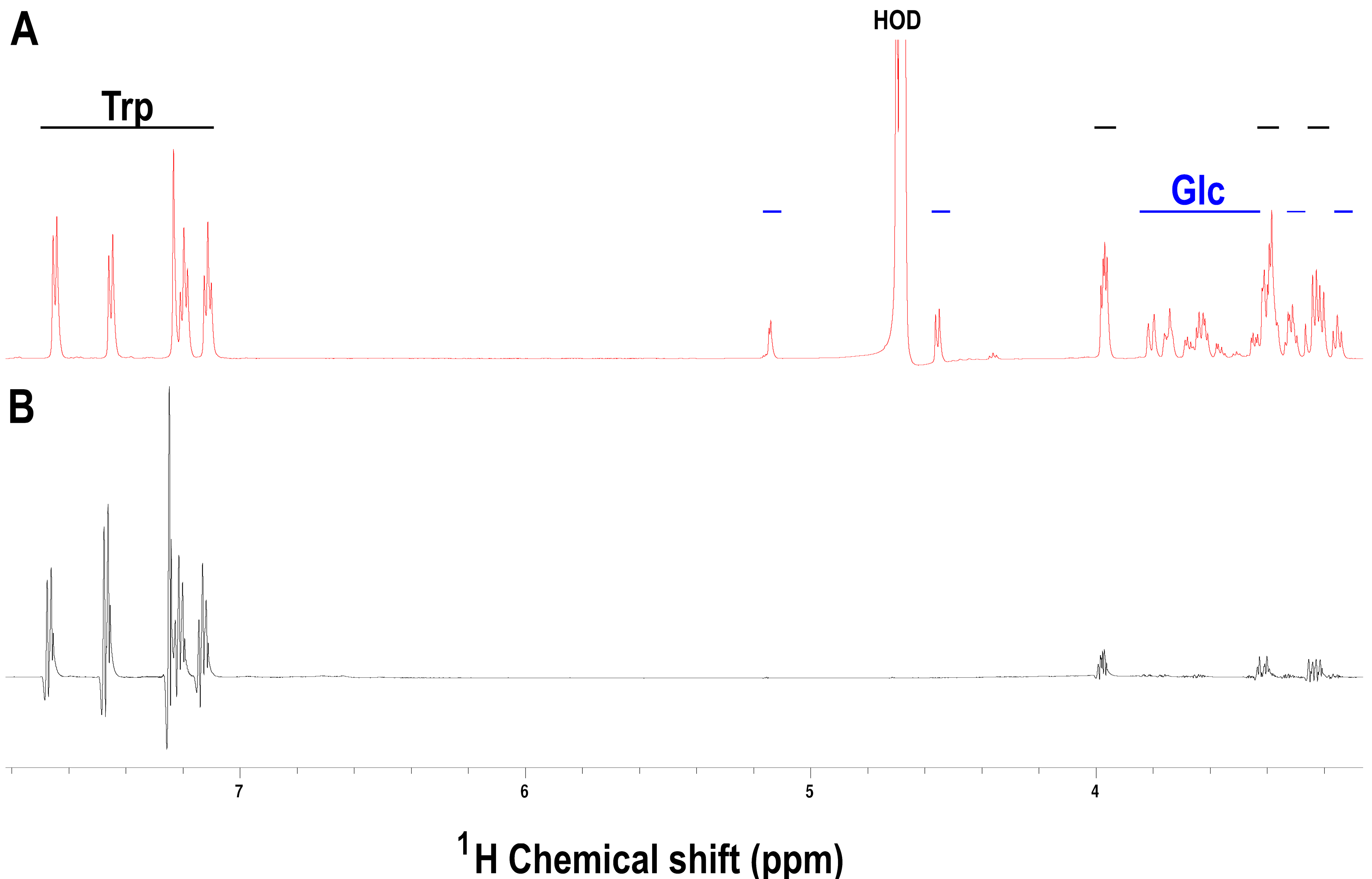

Supplement: S3 Fig — Two 1H-1D spectra are presented for a solution of bovine serum albumin, L-Tryptophan (Trp) and Glucose (Glc). Spectrum (A) is the reference spectrum with attenuation of residual solvent using pre-saturation at the solvent frequency. Spectrum (B) is the STD spectrum calculated from the difference of two spectra with excitation off-resonance (30 ppm) and on resonance (-1 ppm). The positive binding of Trp to BSA is indicated by the presence of signals that have obtained their excitation via the protein lattice. Glc does not bind and so does not register in the difference spectrum. (TIF) [file pone.0128803.s003.tif]

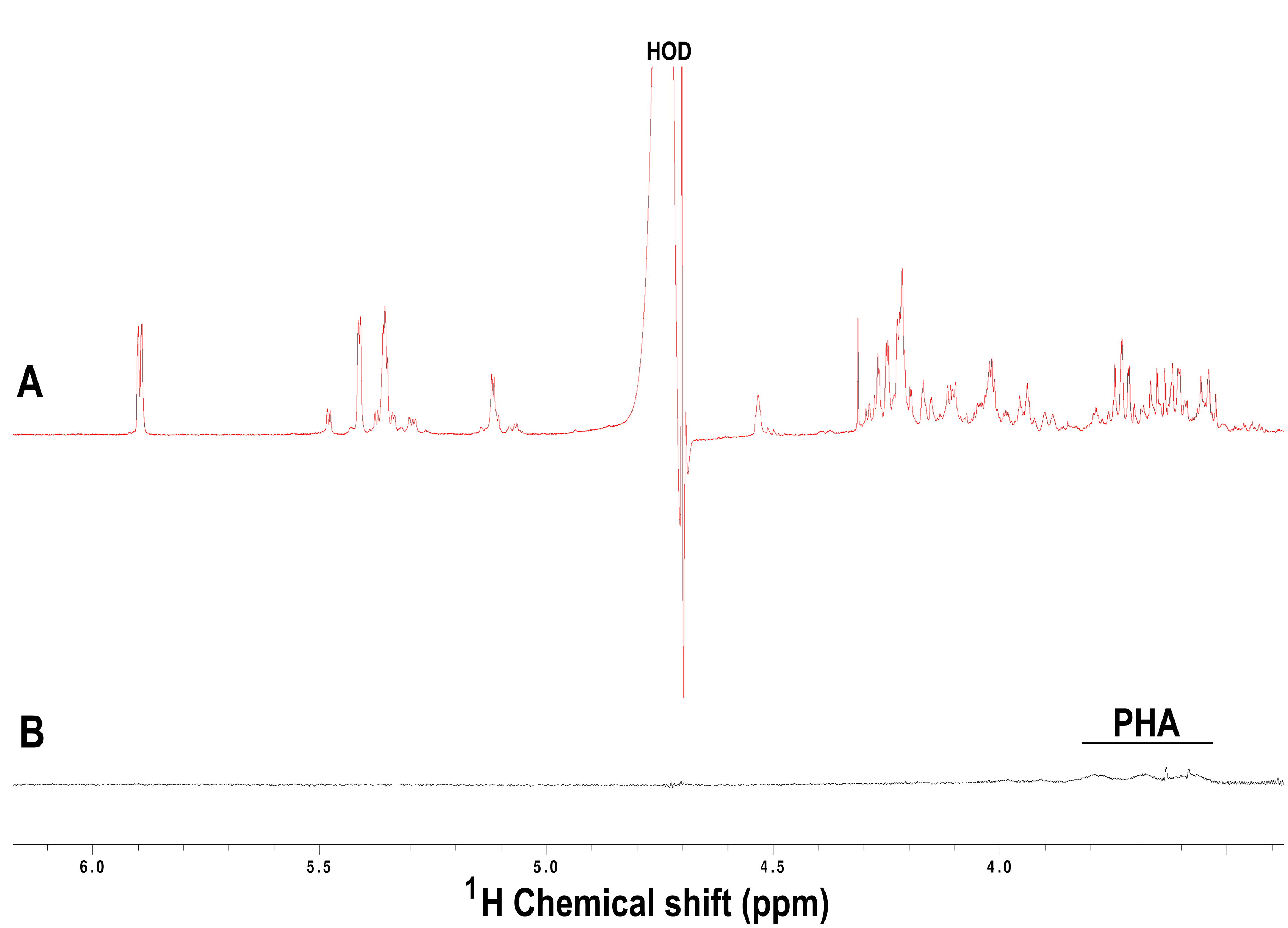

Supplement: S4 Fig — Two 1H-1D spectra are shown for the putative binding of fraction 4 toPHA. Spectrum (A) is the reference spectrum of fraction 4 and PHA obtained with suppression of residual solvent signal by pre-saturation at the solvent frequency. Spectrum (B) is the STD difference spectrum calculated from spectra with an on-resonance pulse at -1 ppm and off-resonance pulse at 30 ppm. Only the baseline of PHA signals can be observed in this trace. No binding of fraction 4 can be detected. (TIF) [file pone.0128803.s004.tif]
